# Nurses in the Male Wards of Asylums for the Insane

**Published:** 1904-07-16

**Authors:** John G. Havelock

**Affiliations:** Sunnyside, Montrose, N.B.


### NURSES IN THE MALE WARDS OF ASYLUMS FOR THE INSANE.

*To the Editor of* THE HOSPITAL.

DR. JOHN G. HAVELOCK writes from Sunnyside, Montrose, N.B.: The communication from an "Old Medical Superintendent" in this week's HOSPITAL recalls to my mind a statement in the Annual Report of Montrose Royal Asylum by Dr. W. A. F. Browne in 1836, in which he mentions with pride that a mirror had been hanging in the Common Hall for the past year and still remained uninjured! Your veteran correspondent naturally, is somewhat out of touch with modern methods of asylum management, and is probably ignorant of the fact that during the last decade the nursing of a certain proportion of male patients by trained female nurses has been carried out in certain asylums with conspicuous success, and a visit to any of these institutions would convince him that the work is neither "revolting" nor "laborious." The difficulties of management, I am assured, have nothing to do with the patients, but are wholly owing to the necessity of having a mixed male and female staff.

